# Psychological Symptom Risks in Spouses of Cancer Patients and Barriers to Seeking Mental Health Counselling: A Descriptive and Correlational Study

**DOI:** 10.1111/scs.70045

**Published:** 2025-06-12

**Authors:** Nazan Turan, Meltem Anafarta Şendağ

**Affiliations:** ^1^ Vocational School of Health Services Gazi University Ankara Turkey; ^2^ Faculty of Social Sciences and Humanities Ankara Social Sciences University Ankara Turkey

**Keywords:** cancer, caregiver, psychological distress, seeking help

## Abstract

**Objective:**

This study aims to examine the risk of psychological symptoms in spouses of cancer patients and the barriers to and determinants of seeking mental health counselling.

**Methods:**

The study was conducted with a descriptive and correlational design. The data were obtained from spouses (*n* = 201) of cancer patients. Data collection involved the Participant Information Form (PIF), Psychological Symptom Screening Test (SCL 90‐R) and Barriers to Seeking Mental Health Counselling Scale (BMHC). Descriptive statistics and regression analysis were used to analyse the data.

**Results:**

Participants exhibited high levels of risk in somatization (1.67 ± 0.86), depression (1.94 ± 0.92), anxiety (1.72 ± 0.68) and additional items (1.55 ± 0.53). In multiple linear regression analysis, the longest place of residence (*β* = −0.137), gender (*β* = −0.144), income level (*β* = 1.152) and depression were associated with BMHC. Additionally, the longest place of residence (*β* = −1.007), gender (*β* = −0.368), income level (*β* = −0.674), somatization (*β* = 0.056), depression (*β* = 0.251) and additional items (*β* = 1.108) were associated with BMHC.

**Conclusions:**

The results showed that despite spouses of cancer patients being at risk of psychological symptoms, they do not seek psychological help due to stigma and lack of knowledge. In addition, the study revealed an important clinical implication that the focus of health services should not only be on the diagnosed cancer patient but also on their spouse.

## 1 Introduction

The World Health Organisation announced that approximately 41 million people die from non‐communicable diseases every year, accounting for 74% of all deaths in the world. In the announcement, it was reported that cancer is the second leading cause of death among the top 10 causes [1]. In the statement made by the International Cancer Research Center, it was stated that one in every five people worldwide has cancer [2]. Therefore, cancers, which cause 9.3 million deaths annually [1], have become one of the most prominent public health issues of the 21st century [3].

A serious illness such as cancer can be a psychologically disturbing experience for the individual, starting from the diagnosis phase until the end of their life [3]. An individual's existential concerns may intensify following a cancer diagnosis [4]. The individual may experience mental health issues such as depression, anxiety, fear of recurrence [5, 6], post‐traumatic stress [7], and may be at risk of suicidal behaviour [8]. This situation may lead to an increase in psychiatric symptoms among cancer patients, adding to their physical symptom burden and posing significant risks to their overall quality of life [9].

Cancer can also cause psychiatric symptoms in caregivers (spouse/romantic partner). Thus, couples are often affected by cancer as a patient‐partner duo and can respond as a whole, employing dual coping [10]. Evidence has shown that spouses experience high levels of anxiety and depression [11], concern [12], stress [13], and sleep disorders [14] during this period. This may negatively affect the quality of support and care spouses provide the patient. Spouses, the most important sources of emotional and social support and care for cancer patients, play critical roles in monitoring and evaluating the patient's symptoms and making care decisions [15]. Therefore, they may often need the assistance of supportive resources and seek help to address the challenges they experience as a couple [16].

Help‐seeking is essential in achieving better support and quality of life for patients and those who care for them. It also encompasses professional behaviours aimed at seeking assistance from healthcare providers for health changes that surpass individual resources and are influenced by various factors [17, 18]. However, studies indicate that many people view seeking professional help as a last resort [18], and individuals may avoid or delay seeking professional help for mental health problems [19].

The literature states that factors hindering individuals' behaviour in seeking professional mental health services generally include lack of awareness of the problem, denial, uncertainty about where to seek help, lack of time and difficulty expressing emotions [20, 21]. Additionally, variables such as age, social and self‐stigma [21, 22], lack of knowledge, cultural understanding and financial barriers can influence individuals' decisions to seek mental health help [23].

### 1.1 The Present Study

There is significant evidence in the literature that spouses of patients with terminal illnesses such as cancer may experience psychological distress and other mental health problems [11, 13]. However, it seems that the reasons why spouses do not seek psychological help despite experiencing psychological problems have not yet been adequately explained. The barriers spouses face in seeking psychological help must be identified, and solutions must be developed. Therefore, spouses can be encouraged to seek psychological help at an early stage, potentially preventing the progression of psychological problems. Achieving improved well‐being in the spouse's mental state can have a two‐way effect, increasing the quality of life for both the spouse and the cancer patient. It has been noted that in the cancer care environment, the health status of a spouse has a bidirectional effect (from the spouse to the cancer patient and vice versa) [24]. In this context, this study aims to examine the risk of psychological symptoms in spouses of cancer patients and the barriers to and determinants of seeking mental health counselling. Research hypotheses are as follows:

1. Spouses of cancer patients experience a high level of psychological symptom risk.
2. Spouses of cancer patients have high scores on barriers to seeking mental health counselling.
3. High levels of psychological symptom risk and other factors (e.g., age and gender) predict barriers to seeking mental health counselling.

## 2 Methods

### 2.1 Study Design

The study was designed as a descriptive and correlational study and was conducted online using the snowballing technique between June and September 2023. The STROBE guidelines were adopted when the study was reported.

### 2.2 Participants and Procedure

The sample size for the study was calculated based on the formula suggested by Tabachnick and Fidell [25] for regression analysis, *N* > 50 + 8 m. According to this calculation, it was determined that the sample size should be 130. Participants were selected based on inclusion criteria, which included (1) being 18 years of age or older and married, (2) volunteering, (3) literate in Turkish and (4) the cancer patient's treatment continued. Foreign nationals were excluded as a criterion because of using a culturally adapted measurement tool to determine participants' barriers to seeking mental health counselling.

**FIGURE 1:**
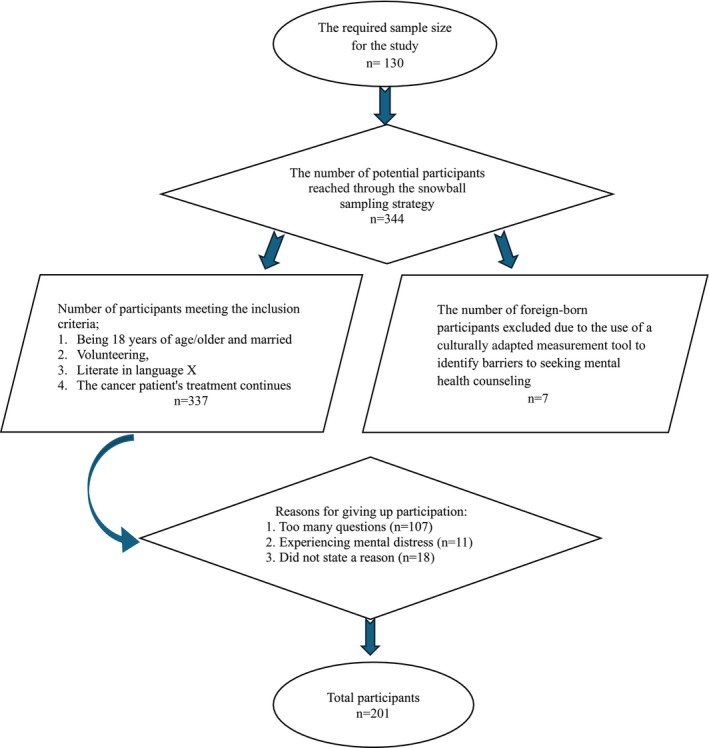
Flow chart.

Nonprobability sampling contained the risk of self‐selection bias. This could pose a significant challenge to the accuracy and reliability of research findings [26]. Additionally, in an online survey, there is no information about who participated, which can lead to the risk of failing to identify the target population [27]. Therefore, cancer patients were contacted first to ensure that all participants were spouses of cancer patients and to enhance the accuracy and reliability of the findings. In this regard, personal and social collaborations were first established. Then, using an online snowball sampling strategy, cancer patients were reached through social media platforms such as Twitter and Facebook, as well as the WhatsApp channels of eight oncology doctors, and an announcement about the research was made. A total of 344 cancer patients responded to the periodically repeated announcement. Subsequently, the spouses of the cancer patients were contacted through them and informed about the research. The participants who met the criteria were administered an online survey consisting of a Participant Information Form, the Psychological Symptom Screening Test (SCL‐90‐R) and the Barriers to Seeking Mental Health Counselling Scale (BMHC), which included 125 questions. The study was completed with 201 participants (Figure 1). All procedures conducted in the study were carried out in accordance with the Helsinki Declaration. Additionally, the study received ethical approval from the review board of University Çankırı Karatekin, and informed consent was obtained from all participants.

### 2.3 Measures

*A participant information form* consisting of nine questions was used to determine the characteristics of the participants, including age, longest place of residence, gender, education level, income level (poverty threshold for a family of four), number of children, partner's cancer stage, need for psychological help and receipt of psychological help.

*The Psychological Symptom Screening Test (SCL‐90‐R)* developed by Derogatis and Cleary [28] was applied to assess the participants' risk for psychological symptoms. This psychiatric screening tool consists of 90 items on a 5‐point Likert‐type scale. It includes 10 sub‐dimensions: Obsessive‐Compulsive, Somatization, Interpersonal Sensitivity, Depression, Anxiety, Anger and Hostility, Phobic Anxiety, Paranoid Ideation, Psychoticism and Additional Items (e.g., item 19, ‘poor appetite’). Each sub‐dimension and the general symptom score are interpreted as follows: 0.00–1.50 is considered ‘normal’, 1.51–2.50 indicates ‘high risk,’ and 2.51–4.00 suggests ‘very high risk’. The Turkish adaptation of the scale, which demonstrated an internal reliability coefficient of 0.97 [29], was used in this study. The internal reliability coefficient of the scale in this study was found to be 0.95.

*The Barriers to Seeking Mental Health Counselling Scale (BMHC)*, developed by Shea et al. [30], assessed participants' barriers to seeking psychological help. This 6‐point Likert‐type scale consists of 26 items and includes six sub‐dimensions: Negative Perceived Value, Discomfort with Emotions, In‐Group Stigma, Lack of Knowledge, Lack of Access and Cultural Barriers. A higher score in each sub‐dimension indicates a more significant perceived disability in that dimension. The scale is scored by averaging the scores of its sub‐dimensions, providing a total score ranging from 27 (lowest) to 162 (highest). In the Turkish adaptation of the scale, the internal reliability coefficient of the total score was 0.87, while for the sub‐dimensions, the coefficients were found to be 0.73, 0.87, 0.85, 0.85, 0.73 and 0.75, respectively [31]. In this study, the internal reliability coefficient of the total score was 0.84, with sub‐dimension coefficients determined as 0.75, 0.78, 0.85, 0.87, 0.76 and 0.80, respectively.

### 2.4 Statistical Analysis

SPSS 26.0 statistical software was utilised for data analysis. Normality plots and skewness/kurtosis statistics were examined to assess the distributions of quantitative variables. Descriptive statistics, including counts, percentages, means and standard deviations, were used to summarise participant characteristics and calculate scores for scale sub‐dimensions and totals. The relationships between scales and their sub‐dimensions were evaluated using Pearson correlation analysis. Univariate and multiple linear regression analyses were conducted to investigate the relationships between the study's dependent variables (SCL‐90‐R and barriers to seeking mental health counselling) and independent variables (longest place of residence, gender and number of children). A significance level of *p* < 0.05 was accepted. The multiple linear regression analysis included independent variables significant in univariate regression analysis. The significance level for the multiple regression was also set at *p* < 0.05.

## 3 Results

### 3.1 Characteristics of Participants

The mean age was 54.12 ± 1.17 years. Of the participants, 71.14% (*n* = 143) were male and 28.86% (*n* = 58) were female. Among them, 75.62% (*n* = 152) resided in urban areas, 24.38% (*n* = 49) lived in rural areas and 88.56% (*n* = 178) had more than one child. Most of the participants had income below the country's poverty line (57.21%, *n* = 115) and (63.18%, *n* = 127) had a high school education. Approximately half of the participants (48.76%, *n* = 98) reported that their partner had stage 3 cancer (breast cancer (20.90%, *n* = 42), prostate cancer (12.44%, *n* = 25), colon cancer (15.42%, *n* = 31)). All participants (100%, *n* = 201) did not receive psychological help, while 94.03% (*n* = 189) expressed a need for psychological help (Table 1).

**TABLE 1:** Characteristics of participants.

Characteristics (*n* = 201)	*n* (%)	Characteristics (*n* = 201)	*n* (%)
Age, years^a^	54.12 ± 1.17 (46–68)^b^	Partner's cancer stage	
Longest place of residence		Stage 1	24 (11.94)
Rural area	49 (24.38)	Breast cancer	9 (4.47)
Urban area	152 (75.62)	Prostate cancer	5 (2.48)
		Colon cancer	2 (1.00)
Gender		Lung cancer	8 (3.99)
Female	58 (28.86)	Stage 2	16 (7.96)
Male	143 (71.14)	Breast cancer	7 (3.48)
Education level		Prostate cancer	3 (1.49)
Primary education	29 (14.43)	Lung cancer	6 (2.99)
High school	127 (63.18)	Stage 3	98 (48.76)
University and above	45 (22.39)	Breast cancer	42 (20.90)
Number of children		Prostate cancer	25 (12.44)
None or one	23 (11.44)	Colon cancer	31 (15.42)
More than one	178 (88.56)	Stage 4	63 (31.34)
Income level		Breast cancer	26 (12.93)
≤ 33.750 TL	115 (57.21)	Prostate cancer	19 (9.45)
> 33.750 TL	86 (42.79)	Colon cancer	18 (8.96)
Needing psychological help		Receive psychological help	
Yes	189 (94.03)	Yes	0
No	12 (5.97)	No	201 (100)

^a^ Mean ± SD.

^b^ Age range (Min‐Max).

### 3.2 Participants' Psychological Symptoms and Barriers to Seeking Mental Health Counselling

The scores of the participants from the SCL 90‐R sub‐dimensions were grouped as 0.00–1.50 (normal), 1.51–2.50 (high risk) and 2.51–4.00 (very high risk). According to SCL‐90 R test results, participants had a high level of risk in somatization (1.67 ± 0.86), depression (1.94 ± 0.92), anxiety (1.72 ± 0.68) and additional items (1.55 ± 0.53) sub‐dimension. Obsessive‐compulsive (1.02 ± 0.65), interpersonal sensitivity (0.75 ± 0.59), anger and hostility (1.03 ± 0.78), phobic anxiety (0.74 ± 0.54), paranoid ideation (1.25 ± 0.70), psychoticism (0.93 ± 0.67) and the general symptom index (1.16 ± 0.69) were found to be within the normal range (Table 2).

**TABLE 2:** SCL 90‐R scores (*n* = 201).

Scale sub‐dimensions	Min	Max	Mean ± SD	Scale sub‐dimensions	Min	Max	Mean ± SD
Obsessive‐compulsive	0.00	2.29	1.02 ± 0.65	Anger‐hostility	0.00	2.78	1.03 ± 0.78
				Phobic anxiety	0.00	2.50	0.74 ± 0.54
Somatization	0.00	3.50	1.67 ± 0.86	Paranoid ideation	0.00	3.54	1.25 ± 0.70
Interpersonal sensitivity	0.00	2.96	0.75 ± 0.59	Psychoticism	0.00	2.81	0.93 ± 0.67
Depression	0.00	3.26	1.94 ± 0.92	Additional items	0.00	3.72	1.55 ± 0.53
Anxiety	0.00	4.00	1.72 ± 0.68	General symptom index	0.05	3.17	1.16 ± 0.69

*Note:* SCL 90‐R: Psychological Symptom Screening Test.

It was found that 2 participants (1.00%) had high‐risk scores in all sub‐dimensions of the SCL‐90‐R, while 135 participants (67.16%) had high‐risk scores in at least one sub‐dimension. A total of 64 participants (31.84%) had scores within the normal range for all sub‐dimensions of the SCL‐90‐R. Participants were determined not to have very high‐risk scores in any SCL‐90‐R sub‐dimensions (Table 3).

**TABLE 3:** Grouping of scores obtained from the SCL‐90‐R sub‐dimensions (*n* = 201).

Scale sub‐dimensions	0.00–1.50 score (normal) *n* (%)	1.51–2.50 score (high risk) *n* (%)	2.51–4.00 score (very high risk) *n* (%)
Obsessive‐compulsive	171 (85.07)	30 (14.93)	0
Somatization	58 (28.86)	143 (71.14)	0
Interpersonal sensitivity	174 (86.57)	27 (13.43)	0
Depression	45 (22.39)	156 (77.61)	0
Anxiety	52 (25.87)	149 (74.13)	0
Anger‐hostility	137 (68.16)	64 (31.84)	0
Phobic anxiety	183 (91.04)	18 (8.96)	0
Paranoid ideation	191 (95.02)	10 (4.98)	0
Psychoticism	198 (98.51)	3 (1.49)	0
Additional items	65 (32.34)	136 (67.66)	0
General symptom index	107 (53.23)	94 (46.77)	0

*Note:* SCL 90‐R: Psychological Symptom Screening Test.

Of the participants identified as high risk, 30 (14.93%) had obsessive‐compulsive disorder, 143 (71.14%) had somatisation, 27 (13.43%) had interpersonal sensitivity, 156 (77.61%) had depression, 149 (74.13%) had anxiety, 64 (31.84%) had anger‐hostility, 18 (8.96%) had phobic anxiety, 10 (4.98%) had paranoid ideation, 3 (1.49%) had psychoticism, 136 (67.66%) had additional items and 94 (46.77%) had general symptom index (Table 3).

Participants' scores on the negative perceived value sub‐dimension of the BMHC scale were 6.48 ± 1.95 (min = 4, max = 24), discomfort with emotions sub‐dimension 7.54 ± 3.56 (min = 5, max = 30), in‐group stigma sub‐dimension 21.47 ± 9.70 (min = 5, max = 30), lack of knowledge sub‐dimension 17.90 ± 6.44 (min = 3, max = 18), lack of access sub‐dimension 5.85 ± 2.51 (min = 4, max = 24) and cultural barriers sub‐dimension 7.71 ± 4.36 (min = 5, max = 30) (Table 4). Since the scale does not have a specific cut‐off score, based on the average scores, participants' scores on the in‐group stigma and lack of knowledge sub‐dimension were high. In contrast, scores on the sub‐dimension of negative perceived value, discomfort with emotions, lack of access, cultural barriers and the total BMHC level can be described as low.

**TABLE 4:** BMHC scores (*n* = 201).

Scale sub‐dimensions	Min	Max	Mean ± SD	Scale sub‐dimensions	Min	Max	Mean ± SD
Negative perceived value	4	24	6.48 ± 1.95	Lack of knowledge	3	18	17.90 ± 6.44
Discomfort with emotions	5	30	7.54 ± 3.56	Lack of access	4	24	5.85 ± 2.51
In‐group stigma	5	30	21.47 ± 9.70	Cultural barriers	5	30	7.71 ± 4.36

Abbreviation: BMHC, Barriers to Seeking Mental Health Counselling.

### 3.3 Factors Associated With Barriers to Seeking Mental Health Counselling

Univariate and multiple linear regression analyses were conducted to examine the impact of participants' characteristics and high‐risk psychological symptoms on BMHC subscales. The established models for negative perceived value, discomfort with emotions, lack of access and cultural barriers, which had low average scores in the BMHC subdimensions, were not found to be significant (*p* > 0.05). Univariate linear regression analysis was conducted to assess the impact of participants' characteristics and high‐risk psychological symptoms on in‐group stigma. Independent variables found to be significant in the analysis—longest place of residence, gender, education level, income level, partner's cancer stage and depression—were included in the multiple linear regression model. The longest place of residence (*β* = −0.137), gender (*β* = −0.144), income level (*β* = −1.152) and depression (*β* = 0.128) were associated with BMHC. According to the established model, gender, education level, income level and depression explained 36.7% of the variance in BMHC (Table 5).

**TABLE 5:** Predictors of the in‐group stigma sub‐dimensions.

Covariates/factors	Simple model		Multiple model		
	*β* coefficient	*p*	*Β* coefficient	*p*	VIF
Longest place of residence (Ref: Urban area)	−0.012	**< 0.001**	−0.137	**0.002**	1.314
Gender (Ref: Male)	−1.006	**0.023**	−0.144	**< 0.001**	1.217
Educational level (Ref: High school)	−0.124	**0.002**	−0.268	0.475	2.719
Number of children (Ref: More than one)	−0.047	0.065			
Income level (Ref: > 33.750 TL)	−1.122	**0.027**	−1.152	**0.002**	1.486
Partner's cancer stage (Ref: Stage 3 and 4)	0.121	0.083			
Somatization	0.357	0.062			
Depression	1.532	**0.034**	0.128	**< 0.001**	1.253
Anxiety	0.041	0.069			
Additional items	1.019	0.093			

*Note: β:* regression coefficient, adjusted^2^: 0.367, *F*: 49.754, *p* < 0.001. Values in bold indicate statistically significant differences (p < 0.001).

Abbreviations: Ref, reference; VIF, variance inflation factor values.

Univariate linear regression analysis assessed the impact of participants' characteristics and high‐risk psychological symptoms on lack of knowledge. Independent variables found to be significant in the analysis—longest place of residence, gender, education level, income level, partner's cancer stage, somatization, depression and additional items—were included in the multiple linear regression model. The longest place of residence (*β* = −1.007), gender (*β* = −0.368), income level (*β* = −0.674), somatization (*β* = 0.056), depression (*β* = 0.251) and additional items (*β* = 1.108) were found to be associated with BMHC. In this model, the combination of longest place of residence, gender, income level, somatization, depression and additional items explained 18.2% of the variance in BMHC (Table 6).

**TABLE 6:** Predictors of the lack of knowledge sub‐dimensions.

Covariates/factors	Simple model		Multiple model		
	*β* coefficient	*p*	*β* coefficient	*p*	VIF
Longest place of residence (Ref: Urban area)	−0.254	**0.031**	−1.007	**0.002**	1.908
Gender (Ref: Male)	−1.962	**0.015**	−0.368	**0.025**	1.762
Educational level (Ref: High school)	−0.857	**0.003**	−0.765	0.584	2.401
Number of children (Ref: More than one)	1.149	0.842			
Income level (Ref: > 33.750 TL)	−0.072	**0.049**	−0.674	**0.002**	1.675
Partner's cancer stage (Ref: Stage 3 and 4)	−1.106	**0.003**	0.742	0.327	2.430
Somatization	0.137	**0.004**	0.056	**0.003**	1.817
Depression	0.104	**< 0.001**	0.251	**0.002**	2.739
Anxiety	1.402	0.096			
Additional items	0.286	**0.003**	1.108	**0.015**	1.633

*Note: β:* regression coefficient, adjusted^2^: 0.182, *F*: 29.801, *p* < 0.001. Values in bold indicate statistically significant differences (p < 0.001).

Abbreviations: Ref, reference; VIF, variance inflation factor values.

## 4 Discussion

This descriptive and correlational study examined the risk of psychological symptoms in spouses of cancer patients and the barriers to and determinants of seeking mental health counselling. The study found that spouses of cancer patients exhibited high‐risk psychological symptoms, while lack of information and in‐group stigmatisation were identified as barriers to seeking help. Furthermore, specific psychological symptoms and sociodemographic characteristics were found to be associated with barriers to seeking mental health counselling and the tested hypotheses were accepted.

First, it was determined that 2 study participants (1.00%) had high‐risk scores in all sub‐dimensions of the SCL‐90‐R, while 135 participants (67.16%) had high‐risk scores in at least one sub‐dimension. Additionally, an analysis of the total scores obtained from all participants revealed that the mean depression and anxiety scores were in the high‐risk range. When this result is evaluated about the sociodemographic characteristics of the participants, it aligns with studies suggesting that a lower quality of life and the psychological symptoms of cancer patient caregivers are significantly associated with gender (male), low‐income levels, living in the same household as the patient, and being a caregiver for an advanced‐stage cancer patient [32, 33, 34]. The high caregiving burden of male spouses, their role in supporting the family, and financial difficulties may increase their risk of anxiety and depression [35, 36].

The study also found a high risk in additional items assessing somatisation, sleep disturbances, and other related problems in spouses of cancer patients. The high risk of somatisation may be related to the increased risk of depressive symptoms among spouses, as depression leads to negative and pessimistic cognitive schemas [37]. Indeed, the heavy caregiving burden, the long‐term dependency associated with caring for cancer patients, and changing social roles may place family caregivers at a higher risk for mental disorders and hopelessness [38]. This situation may unconsciously reveal somatisation as a dysfunctional and maladaptive coping mechanism [39]. On the other hand, sleep disturbance is a common problem among caregivers of cancer patients [40, 41]. In this direction, recent studies have provided evidence that sleep disturbance and/or poor sleep quality are seen in advanced cancer caregivers [42, 43].

Regarding the results on barriers to seeking psychological help, it was determined that the scores for lack of information and in‐group stigma were higher among the spouses of cancer patients compared to other barriers. This result is not surprising, as depression and anxiety are positively correlated with stigma [44], and a high risk of depression and anxiety was found among the spouses in this study. Additionally, the public presentation of psychological symptoms (such as depression) can affect stigma and mental health literacy [45]. The ability to recognise the symptoms and risk factors of mental disorders, seek professional help accordingly, and know available resources, accessible professionals, and treatment facilities are key components of mental health literacy [46]. Thus, although many individuals express an intention to seek professional help for mental health issues or to seek help for themselves when experiencing such difficulties, the actual number of those who follow through with seeking help remains low due to a lack of knowledge in this area [47, 48].

Multiple linear regression analysis also showed that increased levels of in‐group stigma among spouses were associated with depression. Additionally, it was determined that higher levels of lack of knowledge were associated with increased somatization, depression and additional symptoms. Factors that prevent patients suffering from mild to moderate depression, anxiety, sleep, eating, or somatization‐related mental disorders from seeking help may include fear of stigma or a desire to cope with problems without professional help [49]. In this regard, the study results support the literature. Although most of the participants stated that they needed psychological help, none of them indicated that they received help.

In the current study, the results of multiple linear regression analysis revealed that in‐group stigma and lack of knowledge scores, which are barriers to seeking mental health counselling, were significantly associated with the longest place of residence, gender and income level among spouses of cancer patients. Specifically, spouses with lower income, residing in rural areas, and female gender exhibited higher levels of in‐group stigmatisation and lack of knowledge. Females in low‐ and middle‐income countries often require family permission to seek professional help. Additionally, they may face barriers to accessing psychological support due to a lack of knowledge about symptoms, fear of social consequences (e.g., divorce or rejection in marriage), shame associated with cancer and financial constraints. Stigmatising attitudes may also be more pronounced when the identified individual is a woman exhibiting depressive symptoms [50].

## 5 Limitations

There are several limitations to the study. Firstly, conducting the study quantitatively using a survey method may have affected the accuracy of the results. Questions may not be uniformly understood in survey‐based research, and responses are limited to the provided options [51]. Therefore, a mixed‐method approach could provide a more comprehensive understanding of psychological symptoms and barriers to seeking psychological help among spouses of cancer patients.

Secondly, the study did not include cancer patients themselves, which might have influenced the findings, as cancer patients and their spouses are typically mutually affected by the disease as a unit [10]. Consequently, it remains challenging to assess the reciprocal impact between spouses.

Finally, the predominance of male participants in the study may have introduced a significant selection bias, limiting the findings' generalizability. Additionally, collecting data exclusively online may have excluded individuals without internet access or those with low digital literacy. In contrast, reliance on self‐reported data may have increased the risk of response bias. Therefore, future studies with similar objectives could be analysed with a larger population and more detailed tests.

## 6 Conclusion

This study found that spouses of cancer patients are at risk of psychological symptoms but often refrain from seeking psychological help due to in‐group stigmatisation and lack of knowledge. Furthermore, demographic characteristics were associated with in‐group stigma, while demographic factors, somatisation, depression and additional symptoms were linked to a lack of knowledge. Therefore, the study underscores an important clinical implication that the focus of health services should not only be on the diagnosed cancer patient but also their spouse. These implications are important for healthcare professionals because spouses lack a systematic support system to manage their psychological symptoms during the cancer patient's treatment process. Hence, healthcare professionals can organise informative campaigns and workshops addressing psychological symptoms in spouses and provide tailored psychological resources. Additionally, mental health professionals can design group‐based awareness interventions for spouses of cancer patients. This can ensure that dysfunctional thoughts and behaviours related to seeking psychological help are replaced with more functional ones, thereby encouraging spouses to seek psychological support.

## Author Contributions

The conception and design of the study: N.T. and M.A.Ş.; acquisition of data: N.T.; analysis and interpretation of data: N.T.; drafting the article or revising it critically for important intellectual content: N.T. and M.A.Ş.; final approval: N.T. and M.A.Ş.

## Ethics Statement

All procedures performed in the study were in accordance with the 1964 Helsinki Declaration and its later amendments. The study was approved by the Çankırı Karatekin University Review Board (2023/8). Informed consent was obtained from all participants included in the study.

## Conflicts of Interest

The authors declare no conflicts of interest.

## Data Availability

The data are available upon request from the corresponding author.
